# Drug-induced Parkinson-like events: a real-world study from 2004 to the first quarter of 2024 based on FAERS

**DOI:** 10.3389/fphar.2024.1529260

**Published:** 2025-01-03

**Authors:** Ke Wang, Junyan Chen, Mingquan Huang, Xinhao Zeng, Xiaoqun Ren, Xiuqiong Liu, Chao Tao, Liuxuan Yang, Jinlu Shang, Meiling Zhou

**Affiliations:** ^1^ Department of Pharmacy, The Affiliated Hospital, Southwest Medical University, Luzhou, China; ^2^ Department of Clinical Pharmacy, School of Pharmacy, Southwest Medical University, Luzhou, China; ^3^ Department of Cardiothoracic Surgery, Luzhou People’s Hospital, Luzhou, China; ^4^ Sichuan Treatment Center for Gynaecologic and Breast Diseases (Breast Surgery), The Affiliated Hospital of Southwest Medical University, Luzhou, China; ^5^ Department of Pediatric Surgery, The Affiliated Hospital of Southwest Medical University, Sichuan Clinical Research Center for Birth Defects, Luzhou, China; ^6^ Department of Pharmacy, West China Hospital Sichuan University Jintang Hospital, Chengdu, China

**Keywords:** Parkinson-like events, pharmacovigilance, drug safety, antiparkinsonian agents, adverse event, FAERS

## Abstract

**Background:**

Timely identification of drug-induced Parkinson-like events is essential to improve clinical management and enhance patients’ quality of life. However, there is a significant lack of studies addressing these events in real-world settings.

**Methods:**

To bridge this gap, we analyzed adverse event (AE) reports related to Parkinson-like events from the FDA Adverse Event Reporting System (FAERS) database from the first quarter of 2004 to the first quarter of 2024. Our objective was to summarize a list of potential drugs at high risk for Parkinson-like events and their corresponding proportions of AE reports.

**Results:**

As a result, a total of 54,639 AE reports linked to Parkinson-like events involving 1,224 drugs were identified. Among these, carbidopa/levodopa exhibited the highest number of reports, followed by baclofen and pimavanserin. The most frequently reported drug class was antiparkinsonian drugs and psycholeptics, followed by psychoanaleptics. Using two disproportionate analysis methods, the reporting odds ratio and proportional reporting ratio, we found that 136 drugs exhibited positive results in both methods, while 1,063 drugs did not show any positive signals.

**Conclusion:**

This study provides a comprehensive pharmacovigilance analysis of drugs associated with Parkinson-like events, aiming to promote rational drug use and inform clinical practice.

## 1 Introduction

Parkinson-like events refer to a group of common neurologic adverse events (AEs) that are clinically similar to Parkinson’s disease (PD). These events are characterized by motor symptoms such as bradykinesia (Bologna et al., 2020), resting tremor (Reich and Savitt, 2019), and muscle rigidity (Postuma et al., 2015), typically manifested unilaterally or asymmetrically. The main conditions contributing to the development of Parkinson-like events are PD and psychiatric disorders. Unlike Parkinson-like events, PD encompasses both motor and non-motor symptoms (Ryman and Poston, 2020). The non-motor symptoms of PD, which include sleep disorders, anxiety, depression, and cognitive impairment (Hayes, 2019), are treated similarly to psychiatric disorders. Drug-induced Parkinson-like events often occur during the treatment of PD and psychiatric disorders.

The World Health Organization has estimated that by 2040, neurodegenerative diseases will become the second leading cause of death in developed countries, surpassed only by cardiovascular disease-related deaths (Mensah-Kane and Sumien, 2023). Therefore, the safety of medications for Parkinson-like events associated with neurodegenerative diseases warrants thorough scrutiny. Although adequate safety studies are conducted before drugs are marketed, these pre-marketing studies are often limited by the size and duration of the trials and typically do not include high-risk populations. As a result, they may not fully reflect the safety profile of drugs in large-scale populations in the real world, leading to the potential occurrence of serious AEs under certain conditions (Silva et al., 2024). Pharmacovigilance is crucial to reducing the incidence of AEs and effectively preventing them. It involves the scientific detection, evaluation, and prevention of AEs or any other potential drug-induced issues (Lucas et al., 2022). Utilizing real-world data from pharmacovigilance databases to explore and summarize drug risk profiles has become an essential approach in assessing the safety of medications (Raschi et al., 2021). The FDA Adverse Event Reporting System (FAERS), one of the largest pharmacovigilance databases globally, is designed to support post-marketing safety surveillance of drugs and therapeutic biologics (Yin et al., 2022; Shu et al., 2023b). This extensive database offers a valuable opportunity to thoroughly assess drug-induced Parkinson-like events.

By collecting and organizing target AE reports in the FAERS database and introducing disproportionality analysis methods for statistical analysis, we can facilitate risk assessment with the massive data, thereby identifying high-risk medications linked to Parkinson-like events (Hu et al., 2020). Currently, there are few studies analyzing large-scale data on drug-induced Parkinson-like events, and the correlation between clinical medication use and the occurrence of these events remains inadequately explored. To promote the rational use of medications, this study aims to uncover the potential associations between drugs and Parkinson-like events by analyzing all relevant reports in the FAERS database from 2004 to the first quarter of 2024, which will provide valuable insights for future clinical practice.

## 2 Methods

### 2.1 Data source

The data for this study were obtained from the FAERS database, which supports post-market safety monitoring of drugs and therapeutic products (Tian et al., 2022). This database includes AE reports submitted by drug manufacturers, consumers, and healthcare professionals (Omar et al., 2021). Notable for its extensive data volume, diverse information, and public accessibility, the FAERS database has published all AE reports received since 2004 on the openFDA website, with updates provided quarterly. Specific data recorded in the AE reports included patient demographic and management information (DEMO), report source information (RPSR), drug information (DRUG), adverse reaction information (REAC), indications for use/diagnosis (INDI), medication therapy initiation and termination dates (THER), and patient outcome information (OUTC) (Shu et al., 2023a). For this study, we collected data from the FAERS database spanning from the first quarter of 2004 to the first quarter of 2024 and managed by SAS 9.4 software. Following FDA recommendations, we deduplicated the data to ensure accuracy and consistency in our analysis.

### 2.2 Identification of target reports

The preferred terms (PTs) in the FAERS database are standardized medical terminologies coded by the Medical Dictionary for Regulatory Activities (MedDRA), based on a wide range of adverse reaction (ADR) information (Liu et al., 2022). Standardized MedDRA Queries (SMQs) consist of a series of PTs indicative of similar medical conditions, allowing the retrieval and optimization of ADR signal detection and assessment within the MedDRA-encoded database, thus enabling both narrow- and broad-scope searches (Bousquet et al., 2019; Chen et al., 2024). In narrow-scope searches, PTs are closely correlated with the disease, whereas in wide-scope searches, the correlation is relatively weak. To ensure accuracy and specificity in identifying target reports, we employed MedDRA version 27.0 and applied the PTs included in the narrow scope of “Parkinson-like events (SMQ)” to identify relevant AE reports (Supplementary Table S1).

### 2.3 Methods of analysis

The clinical characteristics of patients with Parkinson-like events, including age, gender, indication, outcome, and reporting country, were summarized by descriptive analysis to screen for medications associated with these events. Further analysis was conducted using disproportionality analysis, a data mining algorithm used for quantitatively detecting ADR signals in large pharmacovigilance databases. This method allows for comparison between the frequency of Parkinson-like events associated with a specific drug and the background frequency. Disproportionality analysis relies on the classical two-by-two contingency table (Supplementary Table S2). The two most commonly used methods in this analysis are the reporting odds ratio (ROR) (Peng et al., 2020) and the proportional reporting ratio (PRR) (Evans et al., 2001) (Supplementary Table S3). Signals were considered positive when at least 3 target AEs were reported (a ≥3) and either the lower limit of the 95% confidence interval (CI) for the ROR was >1 or the PRR value was ≥2 with a chi-square (χ^2^) value of ≥4. Reports not meeting these criteria were considered negative. A higher ROR or PRR value indicates a stronger statistically significant association between the suspected medication and Parkinson-like events.

## 3 Results

### 3.1 Descriptive analysis

**FIGURE 1:**
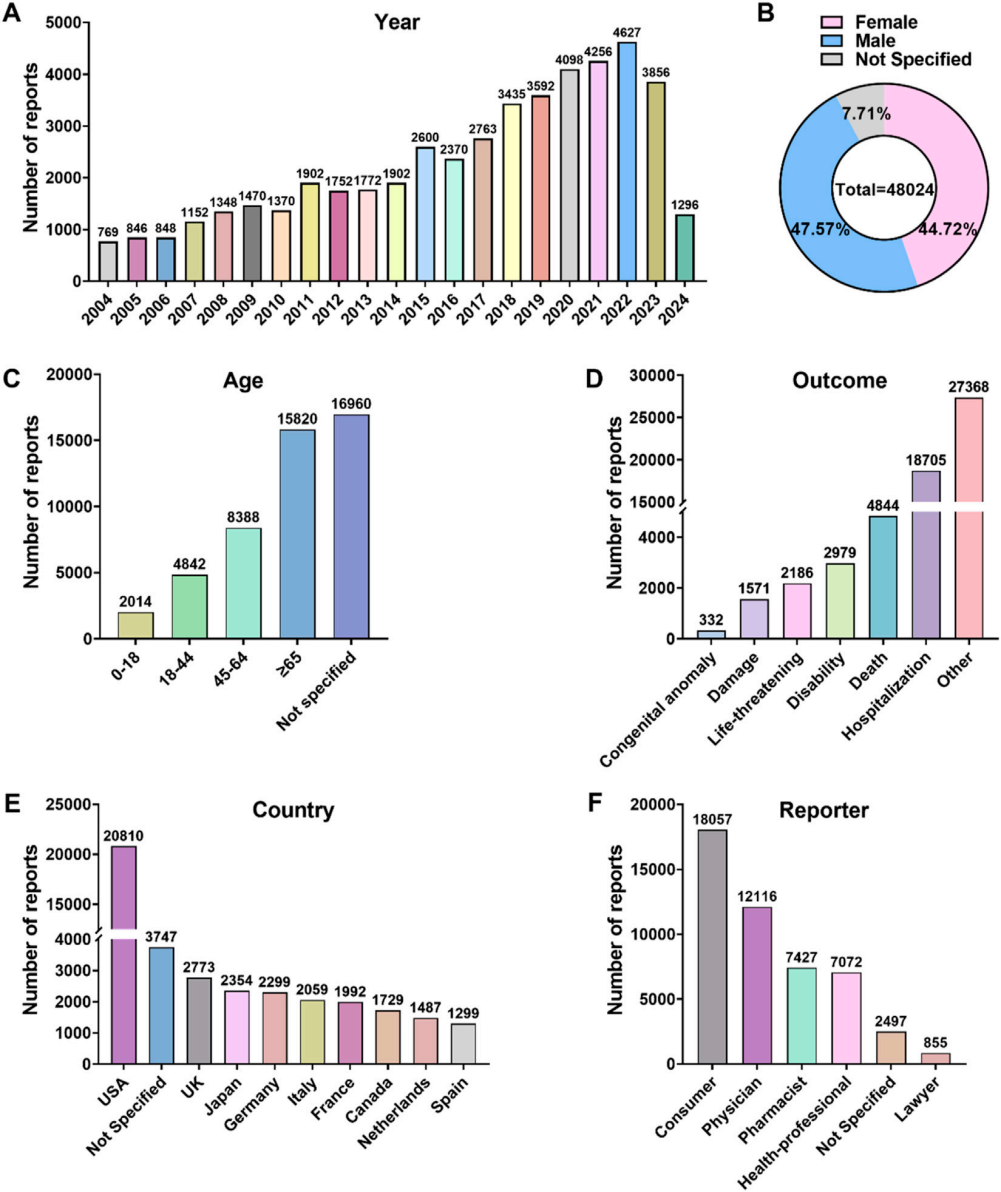
Overview of AE reports associated with Parkinson-like events in the FAERS database spanning from 2004 to the first quarter of 2024. **(A)** Annual AE report counts. **(B)** Patient gender distribution. **(C)** Patient age distribution. **(D)** Distribution of AE outcomes. **(E)** Top 10 countries with the highest number of reports. **(F)** Reporter occupational distribution.

Between the first quarter of 2004 and the first quarter of 2024, a total of 21,161,817 AE reports were included in the FAERS database. After conducting a narrow-scope search for “Parkinson-like events (SMQ)” and removing duplicates, 54,639 reports were identified as related to Parkinson-like events. The annual distribution of these reports is illustrated in Figure 1A, showing a yearly increase in the number of cases, with the highest number of reports, 4,627 cases, received in 2022. In terms of patient gender distribution, Parkinson-like events were slightly more prevalent in males (47.57%) compared to females (44.72%) (Figure 1B). Regarding age distribution, 50.41% of all targeted AEs were reported in patients older than 45 years, with a median age of 65 years (Figure 1C). Hospitalization due to Parkinson-like events accounted for 38.95% of cases, while deaths accounted for 10.09% (Figure 1D). The majority of reports originated from the United States (43.33%) (Figure 1E), and healthcare professionals were the main submitters of the reports, with physicians accounting for 25.23%, pharmacists for 15.47%, and other healthcare professionals for 14.73% (Figure 1F).

### 3.2 Identification of drugs associated with Parkinson-like events

**FIGURE 2:**
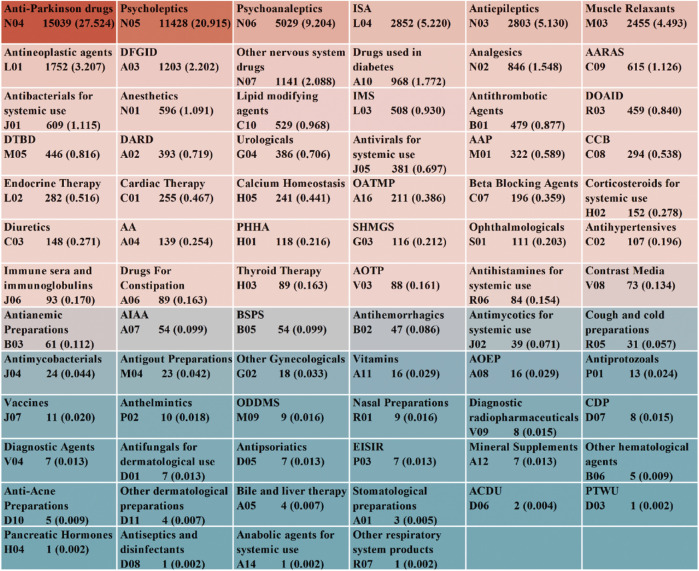
ATC classification and summary of drugs associated with Parkinson-like events. Abbreviations: AARAS, Agents acting on the renin-angiotensin system; IMS, Immunostimulants; DOAID, Drugs for obstructive airway diseases; DTBD, Drugs for treatment of bone diseases; DARD, Drugs for acid related disorders; AAP, Antiinflammatory and antirheumatic products; CCB, Calcium channel blockers; OATMP, Other alimentary tract and metabolism products; AA, Antiemetics and antinauseants; PHHA, Pituitary and hypothalamic hormones and analogues; SHMGS, Sex hormones and modulators of the genital system; AOTP, All other therapeutic products; AIAA, Antidiarrheals, intestinal antiinflammatory/antiinfective agents; BSPS, Blood substitutes and perfusion solutions; AOEP, Antiobesity preparations, excl. diet products; ODDMS, Other drugs for disorders of the musculo-skeletal system; CDP, Corticosteroids, dermatological preparations; EISIR, Ectoparasiticides, incl. scabicides, insecticides and repellents; ACDU, Antibiotics and chemotherapeutics for dermatological use; PTWU, Preparations for treatment of wounds and ulcers.

From the 54,639 targeted AE reports collected, we identified 1,224 medications associated with the occurrence of Parkinson-like events after excluding non-primary suspected medications, duplicate medications, medications with ambiguous or missing generic names, and medications with the same ingredient. These medications were classified using the Anatomic Therapeutic Chemical (ATC) system at the second level, resulting in 76 drug classes (Figure 2). The top 10 drug classes with the highest number of reported cases were antiparkinsonian drugs (n = 15,039), psycholeptics (n = 11,428), psychoanaleptics (n = 5,029), immunosuppressants (ISA) (n = 2,852), antiepileptics (n = 2,803), muscle relaxants (n = 2,455), antineoplastic agents (n = 1,752), drugs for functional gastrointestinal disorders (DFGID) (n = 1,203), other nervous system drugs (n = 1,141), and drugs used in diabetes (n = 968). Further association of these 1,224 drugs with specific PTs, as shown in Supplementary Figure S1, revealed that the top five specific PTs in terms of the number of associated drugs were PD (761), muscle rigidity (742), Parkinsonism (533), hypertonia (519), and bradykinesia (447).

### 3.3 Proportional distribution of drugs in AE reports

**FIGURE 3:**
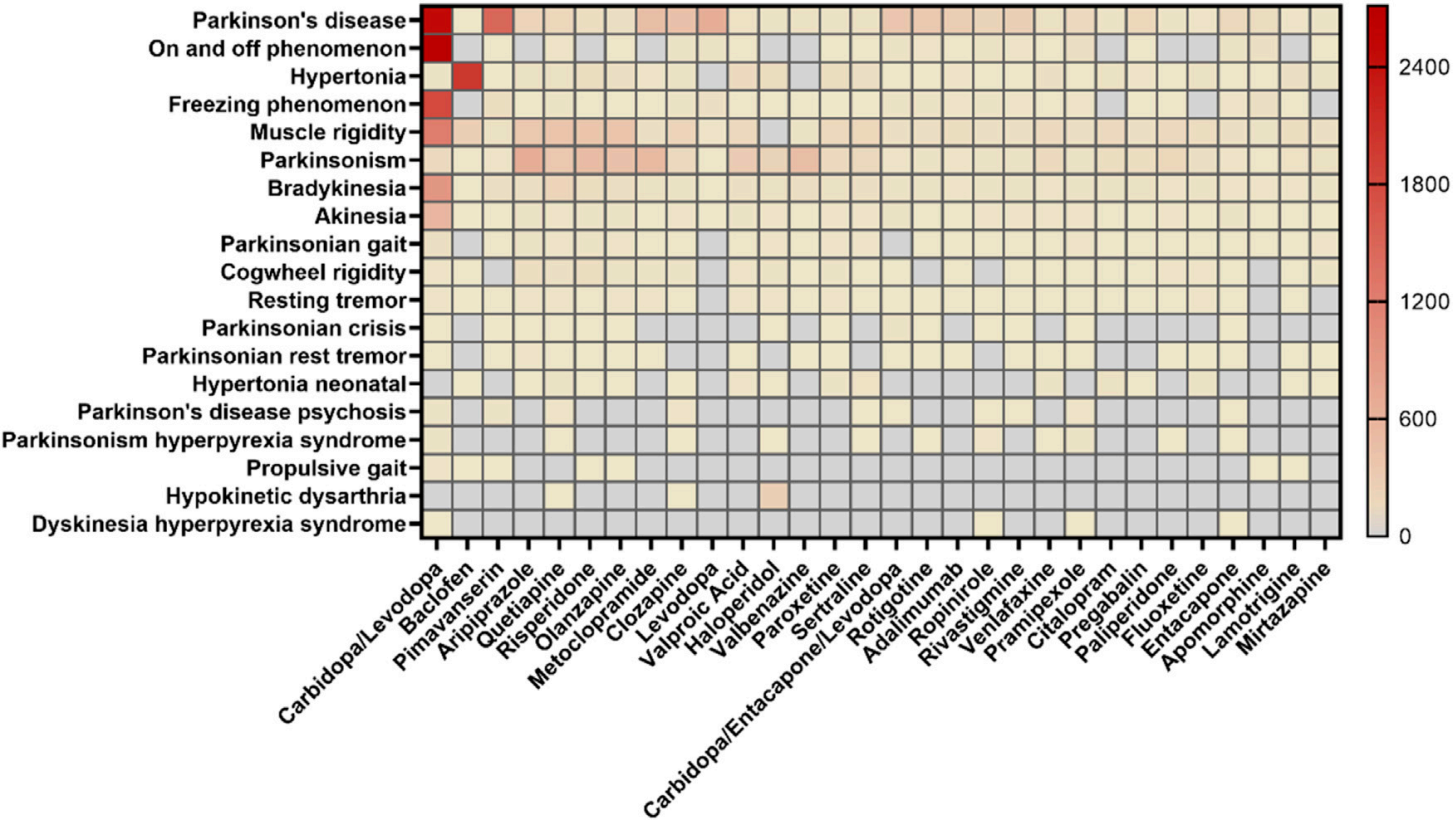
Reporting frequency of top 30 highest agents at the SMQ and PT levels.

**FIGURE 4:**
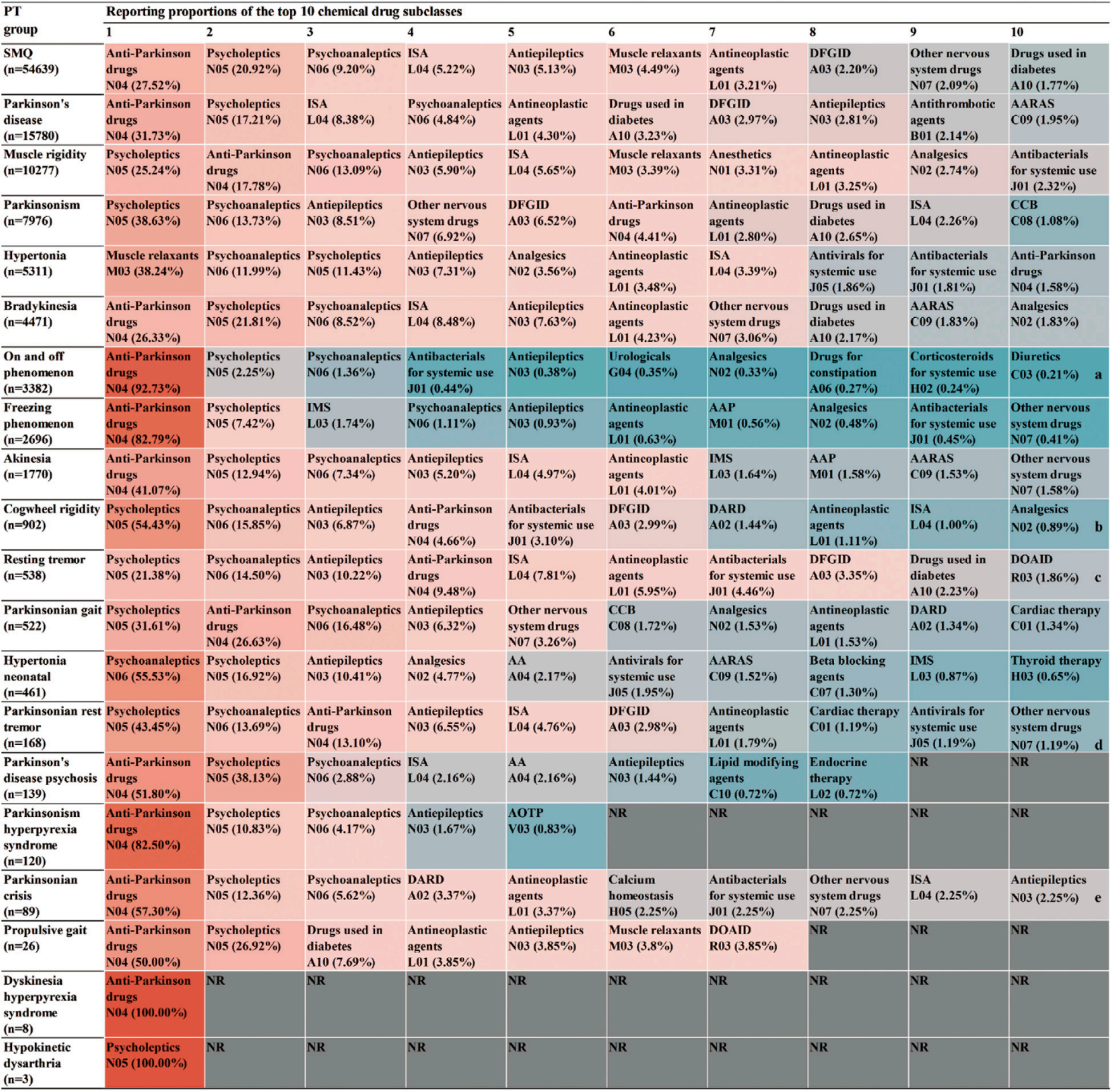
Percentage of reports for the top 10 drug classes at both the SMQ level and the PT level. Abbreviations: NR, no report. a, Antithrombotic drugs (B01) were reported in the same proportion as diuretics (C03). b, Muscle relaxants (M03) were reported in the same proportion as analgesics (N02). c, DARD (A02) and diuretics (C03) were reported in the same proportion as DOAID (R03). d, Urologicals (G04) and antihypertensives (C02) were reported in the same proportion as other nervous system drugs (N07). e, Beta blocking agents (C07) were reported in the same proportion as antiepileptics (N03).

For AE reports related to specific PTs and the drugs they contain, the SMQ levels of the top 30 drugs by frequency of occurrence and the proportional distribution of drugs with different PT levels were calculated separately (Figure 3). In terms of SMQ levels, the top 10 reported drugs were as follows: carbidopa/levodopa (18.48%), baclofen (4.20%), pimavanserin (3.29%), aripiprazole (2.87%), quetiapine (2.50%), risperidone (2.45%), olanzapine (2.20%), metoclopramide (2.17%), clozapine (1.84%) and levodopa (1.46%). Among the top 30 drugs, the most frequently reported were antiparkinsonian drugs (8/30) and psycholeptics (8/30), followed by psychoanaleptics (7/30) and antiepileptics (3/30). DFGID, ONSD, ISA, and muscle relaxants each accounted for 1/30. Further linking the 1,224 medications to specific PTs, variations were observed in the distribution of medications across different PTs, as shown in Figure 4. For instance, for PTs related to Parkinson’s disease, muscle rigidity, and hypertonia, the predominant drug classes reported were antiparkinsonian drugs, psycholeptics, and muscle relaxants, respectively. These findings contrast with the distribution of drugs reported at the SMQ level.

### 3.4 ADR signal detection results

We performed a comprehensive ADR signaling assay on the screening results according to the process in Supplementary Figure S2A to assess the potential risk of Parkinson-like events for the 1,224 drugs screened, and the results are presented in Supplementary Table S4. The top 30 drugs with ADR signaling results are summarized in Table 1, and the top 5 drugs with the highest number of reports were as follows: carbidopa/levodopa (N = 10,096 cases, ROR = 60.48, 95% CI = 59.16–61.83, PRR = 57.56, χ^2^ = 457,905.12), baclofen (N = 2,293 cases, ROR = 22.24, 95% CI = 21.32–23.20, PRR = 21.77, χ^2^ = 43,584.07), pimavanserin (N = 1800 cases, ROR = 18.88, 95% CI = 18.01–19.80, PRR = 18.55, χ^2^ = 28,926.02), aripiprazole (N = 1,568 cases, ROR = 7.43, 95% CI = 7.06–7.81, PRR = 7.38, χ^2^ = 8,407.65) and quetiapine (N = 1,366 cases, ROR = 4.63, 95% CI = 4.39–4.89, PRR = 4.62, χ^2^ = 3,777.60), all exhibiting positive signals. Overall, the results of the disproportionate analysis of ROR and PRR showed that out of the 1,224 drugs associated, 160 drugs were positive for ROR, 137 drugs were positive for PRR, and 136 drugs had positive results for both ROR and PRR (Supplementary Figure S2B).

**TABLE 1:** The ADR signal detection results of the top 30 drugs associated with Parkinson-like events on ROR and PRR.

Drug name	No. of reports	ROR (95% CI)	Signal	PRR	Chi-squared (χ^2^)	Signal
Carbidopa/Levodopa	10,096	60.48 (59.16–61.83)	Y	57.56	457,905.12	Y
Baclofen	2,293	22.24 (21.32–23.20)	Y	21.77	43,584.07	Y
Pimavanserin	1800	18.88 (18.01–19.80)	Y	18.55	28,926.02	Y
Aripiprazole	1,568	7.43 (7.06–7.81)	Y	7.38	8,407.65	Y
Quetiapine	1,366	4.63 (4.39–4.89)	Y	4.62	3,777.60	Y
Risperidone	1,336	5.89 (5.58–6.22)	Y	5.86	5,263.49	Y
Olanzapine	1,203	6.40 (6.04–6.78)	Y	6.37	5,327.05	Y
Metoclopramide	1,183	9.60 (9.06–10.17)	Y	9.52	8,830.49	Y
Clozapine	1,003	3.91 (3.68–4.17)	Y	3.90	2,128.01	Y
Levodopa	795	47.09 (43.83–50.59)	Y	44.96	33,706.26	Y
Valproic Acid	785	5.32 (4.96–5.71)	Y	5.30	2,699.70	Y
Haloperidol	774	19.56 (18.21–21.01)	Y	19.19	13,171.25	Y
Valbenazine	679	16.28 (15.08–17.57)	Y	16.03	9,458.70	Y
Paroxetine	636	3.48 (3.22–3.77)	Y	3.47	1,109.18	Y
Sertraline	627	3.33 (3.08–3.60)	Y	3.32	1,007.09	Y
Carbidopa/Entacapone/Levodopa	597	69.24 (63.69–75.27)	Y	64.68	37,061.06	Y
Rotigotine	546	24.07 (22.10–26.21)	Y	23.51	11,662.34	Y
Adalimumab	491	0.24 (0.22–0.27)	N	0.24	1,150.58	N
Ropinirole	489	19.44 (17.77–21.27)	Y	19.08	8,310.05	Y
Rivastigmine	480	9.24 (8.44–10.11)	Y	9.16	3,461.73	Y
Venlafaxine	466	2.72 (2.48–2.98)	Y	2.71	500.68	Y
Pramipexole	461	14.65 (13.35–16.06)	Y	14.44	5,725.48	Y
Citalopram	437	4.41 (4.01–4.85)	Y	4.40	1,138.13	Y
Pregabalin	436	1.13 (1.03–1.24)	Y	1.13	6.40	N
Paliperidone	424	5.05 (4.59–5.56)	Y	5.03	1,360.72	Y
Fluoxetine	407	4.20 (3.81–4.63)	Y	4.19	980.49	Y
Entacapone	393	55.54 (50.15–61.50)	Y	52.57	19,758.13	Y
Apomorphine	339	15.07 (13.53–16.78)	Y	14.85	4,357.94	Y
Lamotrigine	308	1.58 (1.41–1.77)	Y	1.58	65.61	N
Mirtazapine	307	4.49 (4.02–5.03)	Y	4.48	825.63	Y

Y: yes, positive signal. N: no, negative signal.

## 4 Discussion

Based on the FAERS database, this study conducted a comprehensive evaluation of real-world AE reports of drug-induced Parkinson-like events, describing their basic characteristics. We compiled a list of 1,224 drugs identified as potential risks for Parkinson-like events. Additionally, we quantified the proportion of reports for various drugs and drug classes, and integrated AE signal detection and distribution data for each drug on this list.

The study observed an increase in the number of reports of drug-related Parkinson-like events increased from 769 in 2004 to 54,639 in the first quarter of 2024, indicating an overall upward trend in the number of reports annually. The peak number of reports was observed in 2022 (4,627), with a slight decrease in 2023 (3,856), reflecting a growing awareness of Parkinson-like events among reporters and an improvement in the reporting system in recent years. Geographically, the highest proportion of reports was in North America (47.60%), followed by Europe (31.45%), while Oceania and Africa exhibited lower proportions (1.78% and 0.26%, respectively). These variations may be attributed to differences in healthcare development and infrastructure across continents. Statistical analysis of patient demographics revealed that patients older than 45 years were at risk for Parkinson-like events, which occurred slightly more frequently in men (47.57%) than in women (44.72%), although the difference was not statistically significant. 86.94% of AE reports of Parkinson-like events caused by medications were categorized as severe. The primary outcome was hospitalization (38.95%), with life-threatening or death occurring in 14.64% of cases. This underscores the significant impact of Parkinson-like events on patient safety. It is imperative for reporters to improve their ability to recognize Parkinson-like events for early detection and intervention to minimize the incidence of serious AEs. In conclusion, this study reveals the multidimensional correlation between suspected drugs and the occurrence of Parkinson-like events, emphasizing the need for clinicians to consider possible AEs when prescribing medications and to take timely countermeasures.

The drugs most strongly associated with the occurrence of Parkinson-like events, in terms of SMQ levels, were those used in the treatment of PD, with carbidopa/levodopa being the most frequently reported. Levodopa, a metabolic precursor of dopamine (Haddad et al., 2017), can cross the blood-brain barrier (BBB) and is decarboxylated to produce dopamine, which stimulates postsynaptic dopamine receptors in the striatum, thereby improving the motor symptoms of PD (LeWitt and Fahn, 2016). In contrast, carbidopa serves as a dopamine decarboxylase inhibitor (Hoy, 2019) that reduces the peripheral metabolism of levodopa to dopamine (which cannot cross the BBB), thus enhancing the bioavailability of levodopa within the central nervous system and decreasing the dosage required (Greig and McKeage, 2016). Our data revealed a total of 10,096 Parkinson-like events linked to carbidopa/levodopa. The top five PTs with the highest number of reported cases were the on-off phenomenon (2,713), PD (2,480), freezing phenomenon (1,806), muscle rigidity (1,231), and bradykinesia (938). While carbidopa/levodopa is an important drug in clinical PD management, motor and non-motor fluctuations can emerge as PD progresses (Aquino and Fox, 2015). Motor fluctuations can manifest as early decay off with worsening motor symptoms and “on time” or peak dose dyskinesia, whereas non-motor fluctuations involve abnormal responses, neuropsychiatric issues, and sensory disturbances (Margolesky and Singer, 2017).

The drugs exhibiting the next closest association with the occurrence of Parkinson-like events are psycholeptics and psychoanaleptics, respectively. According to the ATC classification system, psycholeptics include antipsychotics, sedative-hypnotics, and anxiolytics, with antipsychotics representing the predominant subgroup. Antipsychotics are primarily used to treat schizophrenia and other mental disorders characterized by psychotic symptoms. Among antipsychotics, first-generation (typical) agents, such as chlorpromazine, phenazine, thioridazine, haloperidol, and fluphenazine, have been widely used. However, second-generation (atypical) antipsychotics, including pimavanserin, risperidone, quetiapine, aripiprazole, and olanzapine, are now more commonly prescribed due to their improved safety profiles. These second-generation drugs primarily act by blocking dopamine type 2 receptors, which may lead to Parkinson-like events during the treatment of psychosis (Aringhieri et al., 2018). Notably, concomitant psychosis is prevalent among PD patients (Ffytche et al., 2017), and psychiatric abnormalities such as hallucinations and paranoid delusions may occur. Our analysis revealed occurrences of Parkinson-like events associated with antipsychotic drugs such as aripiprazole, quetiapine, and risperidone, which align with previous research by de Germay et al. (2020). Psychoanaleptics, another drug class associated with Parkinson-like events, are known to stimulate the cerebral cortex, medullary respiratory center, and spinal cord, thereby promoting brain function recovery, improving microcirculation, and reducing blood viscosity. Among psychoanaleptics, antidepressants constitute the majority, including commonly used drugs such as venlafaxine, sertraline, fluoxetine, citalopram, and mirtazapine. These antidepressants primarily exert their effects through the modulation of 5-hydroxytryptamine (5-HT) (Ślifirski et al., 2021) and norepinephrine pathways (Khushboo et al., 2022). It has been found that antidepressants increase the stimulation of the 5-HT_2_ receptor by increasing the supply of 5-HT, indirectly inhibiting the release of dopamine in the striatum (Alex and Pehek, 2007), thereby causing Parkinson-like events. A deleterious association has been observed between the occurrence of Parkinson-like events and the use of antidepressants such as mirtazapine, citalopram, paroxetine, sertraline, and venlafaxine. These medications may result in symptoms such as akathisia, tardive dyskinesia, and dystonia (Guo et al., 2018). Our findings are consistent with these reports, highlighting the need to remain vigilant for possible Parkinson-like events when using psycholeptics and psychoanaleptics.

From a pharmacovigilance perspective, although our study provides a comprehensive outlook on medications with potential risks of inducing Parkinson-like events, it is important to recognize certain inherent limitations. Firstly, due to the voluntary nature of the reports collected in the FAERS database, the reporters included individuals without a medical background in addition to healthcare professionals, which may introduce variability and uncertainty in the results. Secondly, various factors such as the patient’s age, drug interactions, dosage, and duration of medication use, and comorbidities can affect the occurrence of Parkinson-like events and the analysis outcomes. Finally, the signal detection results for Parkinson-like events indicate only a statistical correlation between the associated drugs and the target AE reports. Further in-depth studies are required to determine the specific mechanisms and establish a clear causal relationship.

The data collected and analyzed in our study indicate that several drug classes, including antiparkinsonian drugs, psycholeptics, psychoanaleptics, ISA, and antiepileptics, are associated with a high prevalence of Parkinson-like events. Most of these drugs are related to the nervous system. The neurological sensitivity and high incidence of Parkinson-like events underscore the need for clinicians to consider not only the therapeutic benefits of these medications but also their potential AEs to minimize the occurrence and severity of Parkinson-like events. In addition, it is necessary to enhance the awareness of patients and their families regarding Parkinson-like events and to promote rational medication use at different levels.

In this study, we employed the FAERS database, using “Parkinson-like events” as the SMQ, to conduct a multifaceted and multidimensional analysis of drugs that may cause Parkinson-like events. Our aim was to identify possible influencing factors and to enhance our understanding of these events. Our findings indicate that patients with psychiatric disorders and those with underlying Parkinsonian diseases are at particularly high risk for developing Parkinson-like events. Therefore, it is crucial to exercise caution when prescribing therapeutic drugs to these groups. Further research is needed to develop a more rational treatment plan or to develop drugs with a lower incidence of AEs.

## 5 Conclusion

In this study, we collected all AE reports related to Parkinson-like events in the FAERS database from 2004 to the first quarter of 2024 and performed ADR signal detection. We discussed the potential influence of demographic information and summarized the medications that may pose a risk of inducing Parkinson-like events. Our study provides a preliminary overview of the potential risk factors and medications involved in Parkinson-like events in the real world. This information can assist supervisory and regulatory authorities, medical staff, and others involved in medication management to better understand the potential risks and optimize clinical treatment regimens. Since the data are sourced from the FAERS database, which relies on voluntary reporting, reporting bias may be introduced. Further in-depth studies are necessary to verify these associations and establish specific and reliable causal relationships. As research progresses, better control over the occurrence of Parkinson-like events can be achieved through improved understanding of disease mechanisms, optimization of drug formulations, and the development of more reasonable drug administration regimens.

## Data Availability

The original contributions presented in the study are included in the article/Supplementary Material, further inquiries can be directed to the corresponding authors.
